# Negligible exposure to nifurtimox through breast milk during maternal treatment for Chagas Disease

**DOI:** 10.1371/journal.pntd.0007647

**Published:** 2019-08-15

**Authors:** Samanta Moroni, Maria Elena Marson, Guillermo Moscatelli, Guido Mastrantonio, Margarita Bisio, Nicolas Gonzalez, Griselda Ballering, Jaime Altcheh, Facundo García-Bournissen

**Affiliations:** 1 Parasitology and Chagas Service, Buenos Aires Children´s Hospital “Dr. Ricardo Gutierrez”, Multidisciplinary Institute for Research in Pediatric Diseases (IMIPP), Buenos Aires Argentina; 2 Toxicology Area, Biological Sciences Department / PlaPiMu-LaSeISiC, Faculty of Exact Sciences, National University of La Plata, La Plata, Buenos Aires, Argentina; 3 PlaPiMu–LaSeISiC, Buenos Aires Committee for Scientific Research, La Plata, Buenos Aires, Argentina; 4 National Research Council of Argentina (CONICET), Buenos Aires Argentina; Tulane University, UNITED STATES

## Abstract

**Background:**

Treatment with nifurtimox (NF) for Chagas disease is discouraged during breast-feeding because no information on NF transfer into breast milk is available. NF is safe and effective for paediatric and adult Chagas disease. We evaluated the degree of NF transfer into breast milk in lactating women with Chagas disease.

**Patients and methods:**

Prospective study of a cohort of lactating women with Chagas disease. Patients were treated with NF for 1 month. NF was measured in plasma and milk by high performance liquid chromatography (HPLC). Breastfed infants were evaluated at admission, 7^th^ and 30^th^ day of treatment (and monthly thereafter, for 6 months).

**Results:**

Lactating women with chronic Chagas disease (N = 10) were enrolled (median age 28 years, range 17–36). Median NF dose was 9.75 mg/kg/day three times a day (TID). Six mothers had mild adverse drug reactions (ADRs), but no ADRs were observed in any of the breastfed infants. No interruption of breastfeeding was observed.

Median NF concentrations were 2.15 mg/L (Inter quartil range (IQR) 1.32–4.55) in milk and 0.30 mg/L (IQR 0.20–0.95) in plasma. Median NF milk/plasma ratio was 16 (range 8.75–30.25). Median relative infant NF dose (assuming a daily breastmilk intake of 150 mL/kg/day) was 6.7% of the maternal dose/kg/day (IQR 2.35–7.19%).

**Conclusions:**

The low concentrations of NF in breast milk and the normal clinical evaluation of the breastfed babies imply that maternal NF treatment for Chagas disease during breastfeeding is unlikely to lead to clinically relevant exposures in the breastfed infants.

**Trial registration:**

**Clinical trial registry name and registration number:** ClinicalTrials.gov NCT01744405.

## Author summary

It is not known whether Nifurtimox, a drug for Chagas disease, is significantly transferred into breast milk, and no clinical trials were conducted to evaluate this topic. Treatment with nifurtimox is safe and effective in children and newborns with Chagas disease. Treatment of young women before pregnancy prevents congenital transmission of Chagas disease. This is the first study to measure nifurtimox concentrations in breast milk. We found that presence of nifurtimox into breast milk is limited, and that breastfed babies had normal clinical evaluations with no observable adverse events. None of the mothers had to discontinue breastfeeding due to adverse events. The exposure of nifurtimox through breast milk during the treatment of mothers with Chagas disease does not seem to pose significant risks for the breastfed infants.

## Introduction

Chagas disease (CD) or *American trypanosomiasis*, is a parasitic zoonosis caused by infection with *Trypanosome cruzi* of worldwide distribution, endemic to the Americas, predominantly affecting the poor and medically underserved [1, 2, 3]. Most CD patients are asymptomatic during the acute phase, but progress to a chronic phase that if the disease is left untreated can lead to cardiac and/or digestive complications in almost 30% of the patients [4, 5]. More than 10 million people are infected in South and Central America. Most people acquire CD during childhood. If girls are not treated, they can transmit the infection to their babies. Recently, CD has become a global health problem expanding to virtually all regions of the world via immigration, with many cases reported in Europe and North America [2, 6].

The only two drugs available for the treatment of CD are nifurtimox (NF) and benznidazole (BNZ), Both drugs have similar effectiveness and limitations [3, 7]; Their mechanisms of action, pharmacokinetics or toxicokinetics are still unclear, but they have been used since decades nonetheless, in spite of a high risk of toxicity in adults, especially dermatological reactions [3, 7]. However, the reported incidence of adverse drug reactions (ADR) is much lower in infants and children [8,9,10,11].

Most physicians rarely give women treatment for CD during lactation due to a perceived risk of infant exposure to these drugs through breastmilk. On the other hand, discontinuation of breastfeeding to allow for maternal treatment is not advisable, given that breast milk is the ideal food for newborns, as well as a source of multiple benefits [12]. However, in areas with high birth rates and limited access to health care, the postpartum breastfeeding period may be the only, and brief, period of time when a woman has consistent contact with health services, and may be amenable for CD treatment.

The aim of this study was to prospectively study, for the first time, NF transfer into breast milk in a cohort of lactating women with CD in order to clarify the safety of this practise. The secondary objective is to provide support for evidence-based recommendations for the management of CD during lactation.

## Population and methods

### Ethics statement

The study protocol was approved by the Research and Teaching Committee, and Bioethics Committee of the Buenos Aires Children’s Hospital “Dr. Ricardo Gutierrez”. The approval number is 2011-06-22.1LAC. Written informed consent was obtained from all participants. All parents provided informed consent on behalf of all minor participants evaluated in this trial. The protocol was registered in ClinicalTrials.gov (#NCT01744405).

Women with chronic CD who were breastfeeding and their infants were enrolled in this prospective cohort study at the Parasitology and Chagas Service, Buenos Aires Children´s Hospital “Ricardo Gutierrez”, between October 2011 and February 2012. CD diagnosis we performed with at least two independent serological tests for *T*.*cruzi* antibodies, as per routine clinical care.

Exclusion criteria included: None of the included patients were taking any co-medications. Patients with known medical conditions that could affect result interpretation, a positive pregnancy test, history of NF hypersensitivity or previous NF treatment were excluded from the study.

Treatment: Lactating women with CD received 8 to 12 mg/kg/day, TID NF (120 mg NF tablets) p.o. (Lampit, Bayer, El Salvador), for 30 days [13, 10].

A detailed clinical history, physical examination and routine laboratory tests were obtained at diagnosis, at the end of the first week and 30 days into NF treatment. Patients were then followed as per CD treatment and follow up guidelines.

Treatment response was evaluated by *T*.*cruzi* specific real-time Polymerase Chain Reaction (PCR) performed at diagnosis and at the end of treatment [14,15]. Patients were instructed to use contraception during treatment; a pregnancy test was performed before enrolment.

**Diagnosis of congenital Chagas disease**: All infants under 8 months old were monitored for CD using microhematocrit test. Infants with negative parasitemia were later tested by serology at 8 months of age [10]. Children older than 8 months of age were evaluated with two serological tests for *T*.*cruzi* antibodies [10]. Growth and psychomotor development was assessed in children by experienced paediatricians. Pediatric evaluations were performed at days 0, 7 and 30 of maternal treatment, and monthly thereafter for at least 6 months.

### Analytical methods

Breast milk samples (approximately 30 mL) were collected before the start of NF treatment, and on the 7^th^ (+/- 3 days) and 30^th^ (+/- 3 days) day of treatment. Each milk sample was mixed, total volume recorded and an aliquot stored at -20 C° until analysis. Breastmilk lipid content was not measured.

Venous blood was sampled in heparinized tubes, centrifuged at 3,000 g for 10 min and plasma stored at -20 C° and lyophilized prior to analysis.

A high performance liquid chromatography (HPLC) method was used to determine NF concentration in plasma and milk, as described previously [16]. Briefly, plasma samples were deproteinized with 100 μL tricloroacetic acid (30% w/v), vortexed 20 seconds, sonicated for five minutes and then centrifuged at 8,000 rpm for 5 minutes. Supernatants were mixed with 500 μL of ethyl acetate, precipitated with 100 mg of anhydrous sodium sulfate (to a concentration near saturation) and vortexed for one minute. The mixture was centrifuged at 8,000 rpm for 5 minutes and the organic phase of three consecutive liquid/liquid extraction procedures were recovered together and rotoevaporated to dryness at 40°C and 40–80 bars. The residue was resuspended in 250 μL of methanol, vortexed for 20 seconds and centrifuged 2 minutes before injection in the HPLC.

Breast milk samples (1000 μL) were deproteinized by adding 100 μL of trichloroaceticacid (30% w/v), vortexed for 1 minute and sonicated for 10 minutes, after which the samples were filtered through a 0.45 micron membrane by centrifugation at 8,000 rpm for 20 minutes to obtain an ultrafiltrate of breast milk. The ultrafiltrate was directly injected into the HPLC [17,18].

The limit of detection (LOD) and limit of quantitation (LOQ) for plasma and breastmilk were 0.01 mg/L and 0.1 mg/L, respectively.

Milk-to-plasma (MP) ratios were calculated from single milk and plasma concentration measurements. In those patients that had plasma concentrations below the LOQ (but above LOD), a value equal to half the LOQ (i.e. 0.05 mg/L) was imputed in order to provide a realistic estimate of plasma concentrations that would overall avoid under- or overestimating MP ratios in these patients.

Single-point maximum observed milk concentration for each individual was multiplied by 0.15 L/kg/day (i.e. estimated median milk intake for an infant) to yield the absolute infant daily NF dose (in μg/kg/day) that the infant would ingest per day through breastfeeding. The absolute infant daily NF dose was then divided by the weight-normalized maternal NF dose (in μg/kg/day) and multiplied by 100 to estimate the percent Relative Infant Dose (RID) [19,20,21]. In cases where more than one RID estimate was available for the same patient, the highest RID was chosen for the statistical calculations. The RID represents the percentage of the therapeutic dose (usually taken from the maternal dose) that a baby would be exposed during breastfeeding. The NF dose used for calculations (i.e. 10–15 mg/kg/d) is the actual pediatric dose used in clinical practice [10,22].

## Results

Ten women and their 10 babies were enrolled in the study. All mothers were in the chronic CD stage; six of them had acquired the infection in Bolivia, 3 in Argentina and 1 in Paraguay. Median age and weight of the mothers were 28 years (range 17–36 years) and 58, 5 kg (range 52–73 kg), respectively. Median infant age at the start of maternal treatment was 6.8 months (range 1 month-11 months), and median weight 7.6 kg (range 5–9.5 kg). All infants were healthy, within 25^th^ to 95^th^ percentiles for weight and height for their respective ages. Three babies were exclusively breastfed and seven also received solid foods. Median maternal daily dose of NF was 9.82 mg/kg/day (range 8.3–12 mg/kg/day). [Table 1]

**Table 1:** Individual nifurtimox levels in maternal plasma and breastmilk.

Patient ID*	Maternal NF dose (mg/day)	Maternal NF weight-adjusted dose (mg/kg/day)	Sampling times (days after start of treatment)	Plasma		Breast Milk		Infant daily dose (mg/kg)*	Milk/plasma*	Relative infant NF dose (% weight- adjusted maternal dose)*
				Hours after dose	NF concentration (mg/L)	Hours after dose	NF concentration (mg/L)			
*P1*	720	9.8	9	3.4	LOQ	7	9.5	1.42	190	14.54%
*P2*	720	12	8	9.42	0.2	7.3	6.2	0.93	31	7.75%
*P3*	540	9.1	4	5.1	0.2	1	2.3	0.34	11.5	3.79%
*P4*	540	9.6	10	2.15	1.1	8.58	4.6	0.69	4.18	7.19%
*P4*	540	9.6	31	13.05	LOQ	13	1.6	——	——	——
*P5*	540	9,4	9	2.05	0.8	2.58	4.4	0.66	5.5	7.02%
*P5*	540	9,4	31	11.1	0.2	11.15	2.0	——	10	——
*P6*	540	8,3	8	2	1.1	1.5	1.3	0.19	1.18	2.35%
*P6*	540	8,3	31	9.25	LOQ	9.20	0.90	——	18	——
*P7*	540	10	21	11.5	LOQ	11.45	0.90	0.13	18	1.35%
*P7*	540	10	31	11.42	LOQ	11.39	0.70	——	14	——-
*P8*	540	9,7	9	2.30	LOQ	1.25	1.4	0.21	28	2.16%
*P8*	540	9,7	30	9.05	LOQ	9.10	LOQ	——-	——	——-
*P9*	540	10	8	9.30	LOQ	9.25	LOD	——-	——	——-
*P9*	540	10	31	11.15	ND	11.07	LOD	——-	——	———
*P10*	540	10,3	8	1.16	0.30	1.1	2.50	——-	8,33	6.70%
*P10*	540	10,3	31	10.0	LOQ	9.55	4.6	0.69	92	——-
Median**	**540**	**9.75**	**10**	**9.25**	**0.30**	**9.1**	**2.15**	**0.50**	**16.0**	**6,70**
**Inter Quartile Range**		**[9.45; 10]**			**[0,2–0,95]**		**[1,32–4,55]**	**[0.20–0.69]**	**[8,75–30,25]**	**[2,35–7,19]**

*Data from one patient who had both plasma and milk levels below LOD, but later admitted to not taking the medication as prescribed, has been removed from the analysis to avoid confusion

**Whenever 2 measurements were available for the same patient, the highest value was chosen for the estimation of the median, to avoid biasing results by including multiple values from the same patient

ND: Not done

LOQ: Below limit of quantitation

LOD: Below limit of Detection

Six mothers (60%) had adverse drug reactions (ADR) to NF: 4 were mild (1 vomiting and fever, 1 headache and dizziness, 1 eosinophilia and 1 mild leukopenia) and were able to continue treatment, and 2 were moderate (psychomotor agitation and headache) and led to medication discontinuation by patient decision after 9 and 19 days of treatment, respectively. There were no serious ADRs and no infant had to stop breastfeeding. No ADRs were observed in the breastfed infants, nor any changes in their behaviour, weight progress or other effects potentially attributable to NF. All infants were healthy during and after the study, as assessed by paediatricians skilled in the evaluation of paediatric patients with CD.

Breast milk samples, a total of 17, were taken at a median 9.4 days (range 4–21) after start of NF treatment, so that all patients are assumed to have been at steady state for NF plasma concentrations at the time of sampling. Post-treatment breast milk samples were taken within 24 hours after the last dose. Median plasma NF concentration was 0.30 mg/L (9 samples were LOQ) (IQR of samples that were not LOQ, 0.20–0.95 mg/L). Median milk concentration was 2.15 mg/L (IQR 1.32–4.55). Median milk/plasma NF concentration ratio (MPR) was 16 (IQR 8.75–30.25).

Assuming a 150 mL/kg daily milk intake, the estimated median NF daily infant dose was 0.50 mg/kg/day (IQR 0.20–0.69), representing a median RID of 6.70% of the maternal weight-corrected daily dose (IQR 2.35–7.19%).

Among the 10 infants enrolled in the study, 8 turned out not to have congenital CD, as confirmed by serology at 9 months of age; the remaining 2 were diagnosed with congenital CD and were treated accordingly; Both had a serological response and negative conversion of PCR; None of these infants had any medication related ADRs.

Only one mother showed positive qPCR at the end of treatment. The measured NF concentrations for this mother in blood and milk were below LOD. After re-evaluation, this patient admitted to not taking the drug correctly (and therefore her data were left out of the analysis). A new 60 days NF treatment course was started and the qPCR was negative at the end of treatment and during posttreatment follow-up.

## Discussion

CD transmission can take place by contact with the vector (i.e. known as “kissing bugs”), congenitally, and via transfusions or organ transplantation. Every year an estimated 1,300 children are born with congenital CD in Argentina, but less than half are offered access to treatment.

Recently, small outbreaks have also been linked to ingestions of parasite-contaminated food [3,23]. *T*. *cruzi* has rarely been detected in human milk, only in mothers with bleeding nipples during acute CD infection. In a previous study of 21 lactating women, our group found no presence of *T*. *cruzi* in human milk using qPCR [24]. Even though risks for parasite exposure from breastmilk are unclear, they are unlikely to be significant and CD in the mother is not considered a reason to avoid breastfeeding [25,26,27].

In a previous study by our group, we observed limited transfer of benznidazole (the other drug available for CD) into breastmilk, and no significant risks to the infants [24], and Vela et al later confirmed that benznidazole used during postpartum in women with CD had no negative impacts on the breastfed child, suggesting that there is no need to interrupt breastfeeding [28]. Unfortunately, benznidazole is not consistently available in all endemic countries, which led us to study NF during breastfeeding, encouraged by a theoretical pharmacokinetic model that suggested that the transfer of NF into breastmilk was likely to be very limited [29].

In rural Latin America young women may only sporadically interact with the health system except during pregnancy, delivery and the early postpartum period. Also, short inter-pregnancy intervals may leave few opportunities for CD treatment beyond breastfeeding periods. The heretofore lack of data supporting safety of NF during breastfeeding put health care professionals in the uncomfortable position of deciding between supporting breastfeeding or CD treatment for the mother, thus forgoing widespread recommendations to support exclusive breastfeeding, and to treat CD [12, 30]. However, this choice between Chagas disease treatment and breastfeeding implies risks such as losing the opportunity to treat the mother and hopefully prevent congenital infections in future babies, as well as preventing long term cardiac complications in the mother, or, if treatment is chosen over breastfeeding, increased risks of infant diarrhea, infections and other formula-associated problems.

This study describes the first prospective study of NF transfer to breastmilk in CD patients, suggesting that infants’ exposure to NF via breastmilk would amount to less than 5% of the usual infant weight-corrected NF dose (i.e. 10–15 mg/kg/day). This exposure is below the 10% cut-off commonly used as threshold evaluate risk for exposure to maternal drugs during breastfeeding [31,32,33]. Taking into account the known safety of NF in children, observed NF milk concentrations (i.e. ~10 times lower than therapeutic doses) would not be expected to produce exposures associated to infant ADRs or any other risks. Furthermore, treatment with NF is better tolerated in infants and children with CD than in adults [10,34]. No ADRs were observed in the breastfed infants in our study, and careful evaluation by experienced paediatricians found no behavioural, growth or weight impacts potentially attributable to NF. The potential difficulties of detecting adverse events in children and infants (especially central nervous system events in small infants) have not escaped our attention. However, even if specific ADRs may be hard to pinpoint (e.g. headache), these events do have detectable manifestations that trained pediatricians can detect. Our group also participated in a multidisciplinary study in children using NF to treat Chagas disease and an incidence of 19% of NF related ADRs were observed, the most common being weight decrease, decreased appetite, headache and rash. All ADRs were readily identified by the pediatricians evaluating these children, many of which participated in this study. The overall observed incidence of ADRs in adults in our cohort (60%) is in agreement with the rate previously described in adults [37,13].

Transfer of drugs into breastmilk is a function of molecular weight (MW) and maternal plasma level [31,32,35]. NF is a small molecule (MW = 287) with high oral bioavailability and moderate plasma protein binding (50%) [31]. Our results show clear evidence that the milk concentrations are a function of the plasma concentrations (Table 1). These results follow the general rule stating that drug concentration in human milk are usually low and will seldom lead to levels that could produce a pharmacological response in the nursing infant [31,36]. The MP ratio estimates in our patients was hampered by the fact that many plasma concentrations were below LOD (i.e. detectable but not measurable), thus forcing us to estimate a concentration in order to calculate MP ratios. We chose the value of 50% LOQ (i.e. the median plasma level that is detectable but not measurable) as a good overall estimate for the observed but non-measurable NF concentrations. MP ratios are intended to provide a general (over) estimate of drug transfer into breastmilk for medications taken by the mother, but contain limited information to judge potential exposure of the baby through breastmilk. MP ratios are, in fact, not generally the preferred estimator of potential for infant drug exposure if other, better; indicators of degree of exposure risk are available such as RID. There is an abundance of examples in the literature of drugs that have high MP ratios but negligible infant exposures due to very low milk concentrations [37]. In the case of NF, the median MP ratio of 16 suggests a significant accumulation of NF in breastmilk. Many potential explanations can account for this, but the main possible reason is that NF is a substrate of breast cancer resistance protein (BCRP), which may be responsible for actively transferring it into the breast (and other tissues)^35^. One patient (P1, Table 1) had an estimated MP ratio of 190. This large MP ratio may be related to BCRP polymorphisms, or other factors. Unfortunately, we do not have enough data to explore this interesting observation further [32].

Given the nature of the design of this study (e.g. in many cases, mothers expressed milk at home and brought it to the clinic the next day), we cannot ascertain whether fore or hind milk was obtained in most occasions, as the main objective was to obtain leftover milk and in no way interfere with infants’ breastfeeding. NF concentrations do not vary significantly depending on fat content, and therefore we did not expect to see much variation between hind and fore milk.

A limitation of this study is the small number of infants enrolled, which makes it impossible to rule out uncommon ADRs. However, relatively large numbers of paediatric CD patients, including infants and neonates, have been treated with NF at therapeutic doses (approximately 8 to 10 times higher than the expected exposure through breast milk based on our data) for the past few decades in many centres in Latin America, and no significant developmental problems or other significant ADRs have been identified to date [10,38,39,29]. We have no reason to believe that a significantly lower exposure would lead to ADRs not observed at therapeutic doses.

## Conclusion

The results of this study, the first of its kind in CD, suggest that NF may be compatible with breastfeeding due to limited drug transfer into breast milk, and low overall infant exposure. The currently perceived contraindication to NF treatment during lactation, so far unsubstantiated by any evidence, may lead to lost opportunities to treat lactating women. This conclusion is further supported by the complete absence of ADRs attributable to NF in the breastfed infants. Our study provides, for the first time, support for continuation of breastfeeding during maternal CD treatment with NF, a practice that can potentially benefit many women and their breastfed infants in settings where maternal treatment during breastfeeding may be advantageous.

## Supporting information

S1 Checklist STROBE statement—Checklist of items that should be included in reports of *cohort studies*. (DOC)
